# An optimal ubiquitin-proteasome pathway in the nervous system: the role of deubiquitinating enzymes

**DOI:** 10.3389/fnmol.2014.00072

**Published:** 2014-08-19

**Authors:** Gorica Ristic, Wei-Ling Tsou, Sokol V. Todi

**Affiliations:** ^1^Department of Pharmacology, Wayne State University School of MedicineDetroit, MI, USA; ^2^Department of Neurology, Wayne State University School of MedicineDetroit, MI, USA

**Keywords:** brain, glia, neuron, neurodegeneration, protease, proteasome, synapse, ubiquitin

## Abstract

The Ubiquitin-Proteasome Pathway (UPP), which is critical for normal function in the nervous system and is implicated in various neurological diseases, requires the small modifier protein ubiquitin to accomplish its duty of selectively degrading short-lived, abnormal or misfolded proteins. Over the past decade, a large class of proteases collectively known as deubiquitinating enzymes (DUBs) has increasingly gained attention in all manners related to ubiquitin. By cleaving ubiquitin from another protein, DUBs ensure that the UPP functions properly. DUBs accomplish this task by processing newly translated ubiquitin so that it can be used for conjugation to substrate proteins, by regulating the “where, when, and why” of UPP substrate ubiquitination and subsequent degradation, and by recycling ubiquitin for re-use by the UPP. Because of the reliance of the UPP on DUB activities, it is not surprising that these proteases play important roles in the normal activities of the nervous system and in neurodegenerative diseases. In this review, we summarize recent advances in understanding the functions of DUBs in the nervous system. We focus on their role in the UPP, and make the argument that understanding the UPP from the perspective of DUBs can yield new insight into diseases that result from anomalous intra-cellular processes or inter-cellular networks. Lastly, we discuss the relevance of DUBs as therapeutic options for disorders of the nervous system.

## Introduction

The Ubiquitin Proteasome Pathway (UPP) is responsible for degrading the majority of proteins in eukaryotic cells. The UPP is important to all steps and processes of the nervous system, including cell fate specification, differentiation, migration, networking, and maturation, and is critical in maintaining neuronal homeostasis during ageing. As post-mitotic cells, neurons cannot disperse toxic or misfolded proteins through cell division, but must instead continuously rid themselves of cellular components whose accumulation could be detrimental. The importance of the UPP to the ageing nervous system is exemplified by neurodegenerative disorders such as Alzheimer's, Parkinson's and other diseases, where pathological hallmarks are the accumulation and aggregation of proteins with toxic properties.

**Figure 1:**
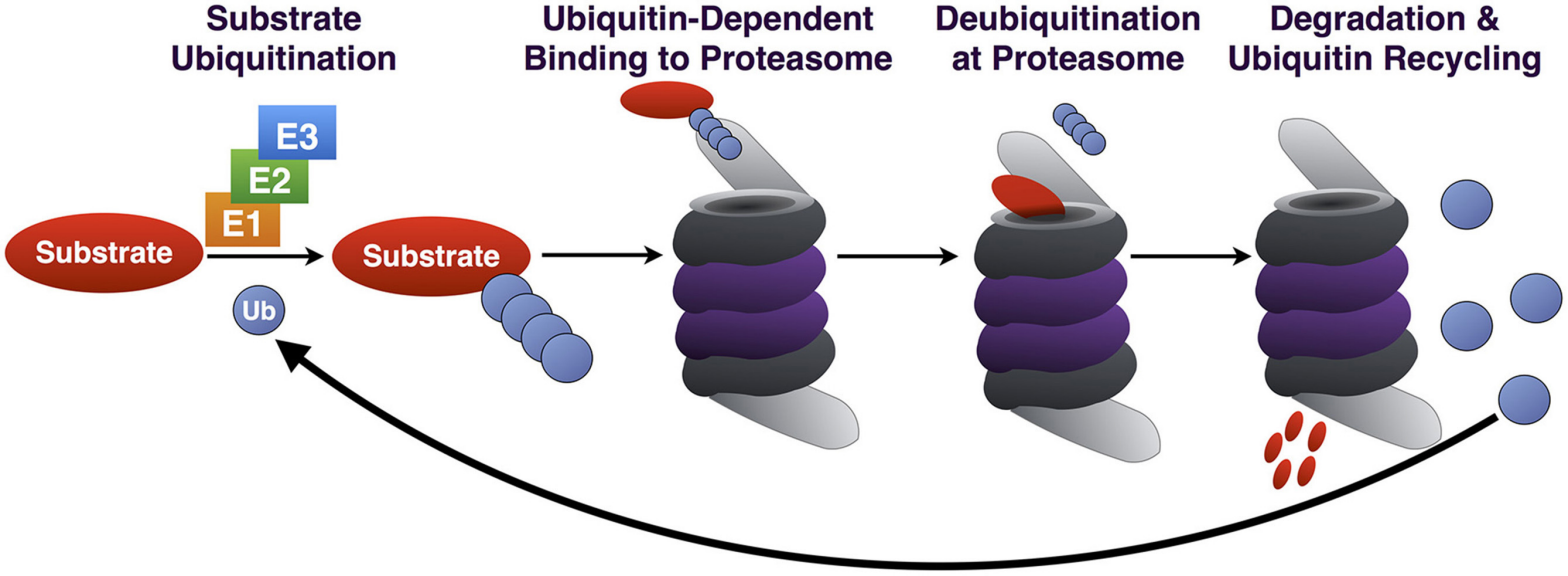
**Summary of the UPP**. Through the coordinated action of E1/E2/E3, Ub is conjugated to a substrate destined for proteasomal degradation. Ub-binding proteins that are part of the 19S or that associate with it reversibly (not depicted) bind poly-Ub on the substrate, which is subsequently deubiquitinated, unfolded and degraded. Poly-Ub is then processed into mono-Ub which re-enters the cycle.

Degradation of proteins by the UPP is a highly selective process. In order for proteins to be degraded by the proteasome, they require a “tag” to identify them as substrates. This “tagging” task is accomplished through the post-translational modification of substrates with the small modifier protein ubiquitin (Ub). Ubiquitination involves the covalent attachment of Ub via an isopeptide bond to a lysine residue of target proteins through the coordinated action of a Ub activating enzyme (E1; two such enzymes are known in mammals), a Ub conjugating enzyme (E2; ~50–75 in mammals), and a Ub ligase (E3; >500 in mammals) (Figure 1).

Ub can be added onto a protein as a monomer or as a poly-Ub chain. Poly-Ub can take on different topographies due to the presence of seven lysine residues on Ub itself, creating chains of different linkage types: K6, K11, K27, K29, K33, K48, and K63. Different types of chains signal different outcomes. In the case of UPP, K48-linked chains consisting of at least four Ub most commonly identify a protein as a proteasome substrate (Thrower et al., 2000; Komander and Rape, 2012; Heride et al., 2014). K11 chains have also been implicated in protein degradation during mitosis (Jin et al., 2008), although it is not clear how widely these chains are utilized by the UPP in other cellular processes.

The proteasome is able to bind both K63- and K48- linked chains in reconstituted systems *in vitro* (Kim et al., 2007; Peth et al., 2010). However, in mammalian cells there appears to be selectivity for K48-linked poly-Ub by the proteasome (Nathan et al., 2013). K63-linked poly-Ub chains are bound by proteins involved in the Endosomal Sorting Complex Required for Transport (ESCRT) pathway, which do not function directly with the proteasome. Binding of K63-linked poly-Ub by ESCRT proteins seemingly precludes the proteasomal degradation of proteins modified with this type of chain (Nathan et al., 2013). Additional specificity in poly-Ub recognition is provided by a family of proteasome-associated proteins, including the Rad23 orthologs hHR23A and hHR23B that selectively bind K48-linked chains (Nathan et al., 2013).

The 26S proteasome is a macromolecular structure composed of a catalytic 20S subunit and one or two 19S regulatory subunits. Ubiquitinated substrates are recognized by and bind to the 19S particle. This process is accomplished in part by the integral 19S receptors S5a and Adrm1. Ubiquitinated proteins that are bound by the 19S proteasome are deubiquitinated and unfolded. The unfolded proteins can then pass through the hollow, cylindrical core of the 20S particle, where they are enzymatically degraded.

Erroneous ubiquitination of a protein could send it to the proteasome prematurely, or could target it for the wrong pathway (e.g., autophagy rather than the UPP), leading to unintended consequences for cells. Specificity for which proteins are ubiquitinated and the type of Ub linkage formed rests in large part with the E2/E3 pair that performs the ubiquitination process (Komander and Rape, 2012; Heride et al., 2014). An additional level of control is provided by enzymes known as deubiquitinases (DUBs), which reverse the isopeptide bond and thus help to control the status of protein ubiquitination. An increasing number of reports is being published on the role of DUBs in nearly all cellular pathways, tissues and organs, in normal homeostasis and in various diseases, including disorders of the nervous system (Todi and Paulson, 2011; Clague et al., 2013).

**Figure 2:**
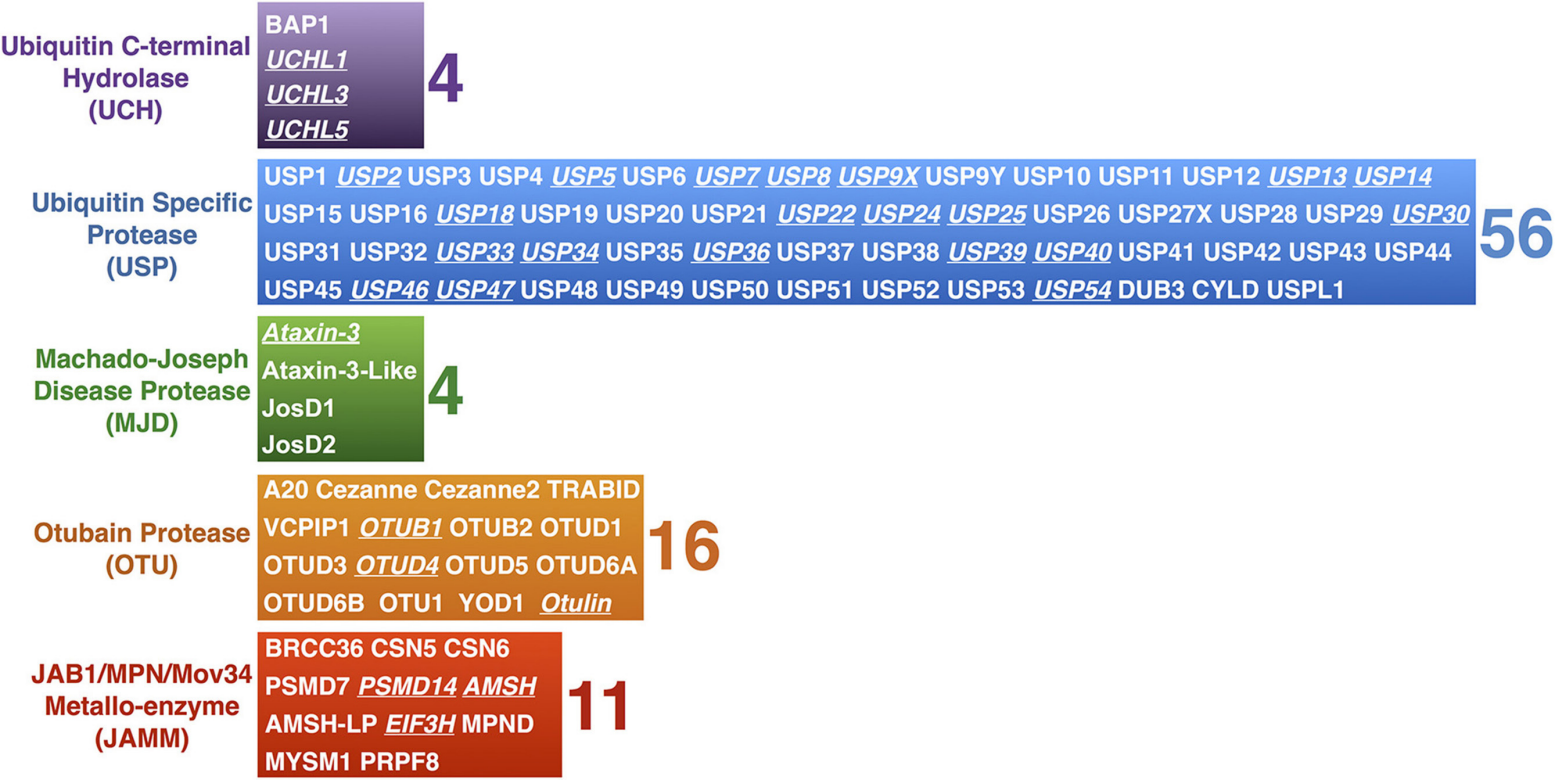
**Mammalian DUBs**. Mammalian DUBs categorized by similarities at the protease domain. Highlighted are DUBs involved in the nervous system. With the exception of JAMM proteases, which are zinc-dependent metallo-proteases, the rest are cysteine proteases.

**Figure 3:**
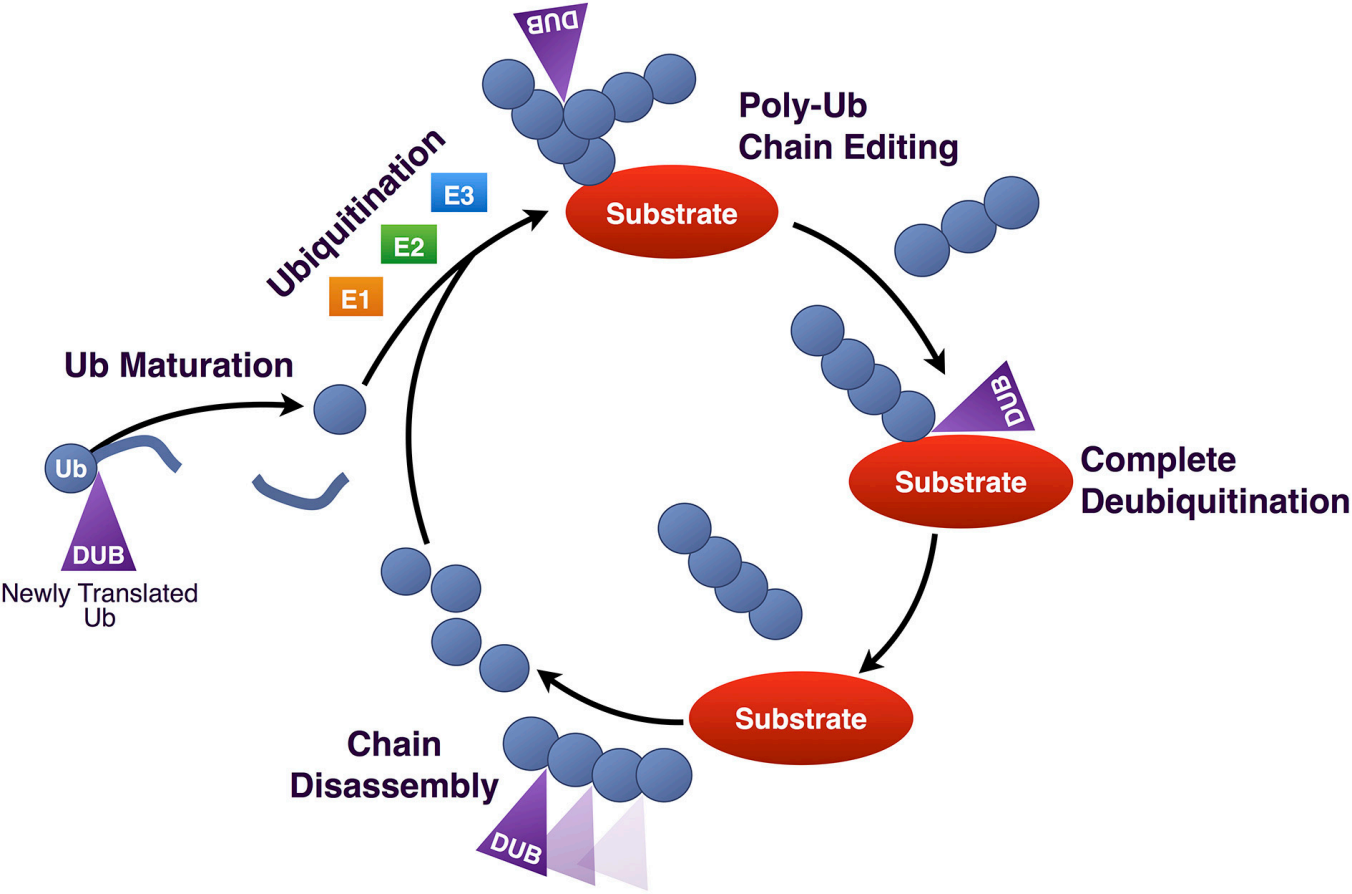
**Roles of DUBs in Ub homeostasis**. Diagrammatic summary of the various functions of DUBs in Ub homeostasis. Newly produced Ub is C-terminally fused to other proteins, such as other Ub molecules, and must be cleaved by DUBs in order for its terminal glycine residue to be exposed for isopeptide bond formation. Once conjugated to a substrate protein, poly-Ub chains can be edited, or can be completely removed. Poly-Ub chains that are unanchored are processed into single Ub and re-enter the Ub conjugation cycle.

The nearly 95 DUBs that are encoded by the human genome are subdivided into five categories based on homology at the catalytic domain. The Ubiquitin C-terminal Hydrolases (UCH), Ubiquitin Specific Proteases (USP), Machado-Joseph Disease Proteases (MJD), and Otubain (OTU) Proteases are cysteine proteases, while JAB1/MPN/Mov34 Metallo-enzyme (JAMM) proteases are zinc-dependent metallo-proteases (Figure 2). DUBs maintain the cellular pool of mono-Ub available for conjugation by processing Ub precursors; they replenish mono-Ub by cleaving poly-Ub chains and recycling Ub; they can fully deubiquitinate substrates and reverse their outcome; or they can edit poly-Ub chains on substrates to help direct them toward a specific pathway (Figure 3). Although at the most basic level catalytically active DUBs perform a similar function—disassembly of Ub-protein bonds—*in vitro* and *in vivo* studies have collected evidence that these proteases have various non-redundant roles (Clague et al., 2013). Distinct roles stem in part from differences in the structure of the catalytic domains of DUBs and in part from interaction domains and subcellular localization signals encoded in their amino acid sequences.

**Figure 4:**
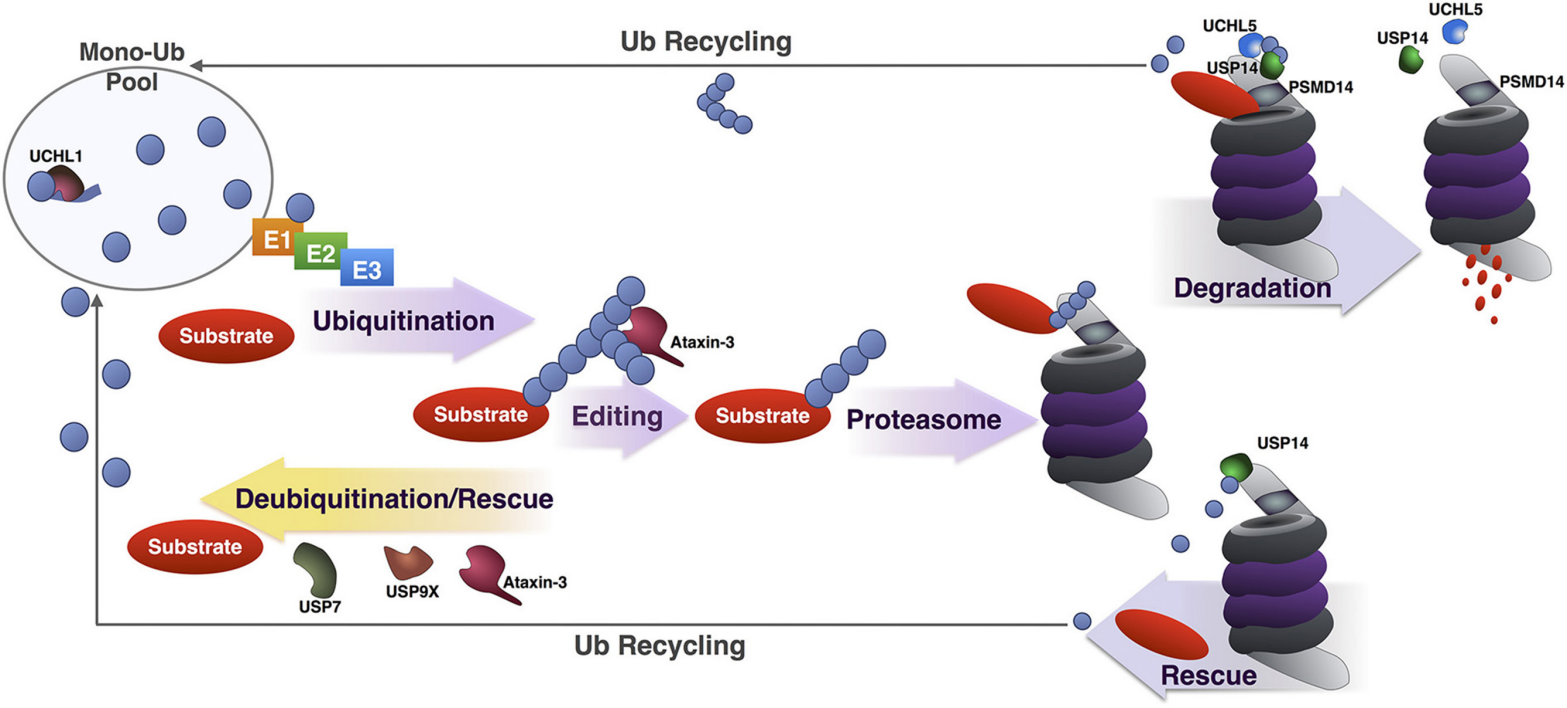
**Summary of DUBs involved in various steps of the UPP**. Summary of the various steps of substrate ubiquitination and degradation during which specific DUBs involved in the UPP are reported to function. UCHL1 maintains a pool of mono-Ub for conjugation by removing additional amino acids present in newly translated Ub, or by processing accidental thiol or amine modifications formed during Ub reactions. Once a substrate has been ubiquitinated, DUBs such as USP7, USP9X and under some circumstances, ataxin-3 can deubiquitinate and rescue it from proteasomal degradation. Ataxin-3 has also been reported to enhance the degradation of a few substrates by editing poly-Ub chains for better recognition by and access to the proteasome. Once at the proteasome, premature deubiquitination of substrates by USP14 can prevent degradation. If a proteasome-bound substrate has been committed to degradation and is being unfolded, deubiquitination by PSMD14, UCHL5, and USP14 recycles Ub.

Three DUBs associate directly with the proteasome: PSMD14, USP14, and UCHL5. PSMD14 is a stoichiometric component of the 19S regulatory subunit, whereas USP14 and UCHL5 associate transiently with it during protein degradation. Several other DUBs function in conjunction with the UPP at steps that precede the proteasome (e.g., during substrate ubiquitination) or following substrate binding to the 19S (e.g., during ubiquitin chain disassembly and ubiquitin recycling; Figure 4). The following sections provide details on UPP-related DUBs in the nervous system, with examples from each sub-class of this family. The exception are OTU proteases, for which there are few mechanistic data relating them to the UPP in the nervous system. Information that has been reported so far on OTUs is included in Table 1, which is a comprehensive list of DUBs implicated in the nervous system, and also includes proteases that do not necessarily function through the UPP.

**Table 1:** **DUBs in the nervous system**.

**UCH**		
UCHL1	Lack of UCHL1 causes gracile axonal dystrophy in mice. Mutations have been linked to PD and other diseases. An N-terminal truncation of this DUB may prevent PD-like damage in cultured cells, potentially by reducing oxidative stress. Primary function of this DUB appears to be mono-Ub maintenance.	Larsen et al., 1998; Setsuie and Wada, 2007; Bilguvar et al., 2013; Kim et al., 2014
UCHL3	Mutations in mice cause learning and working memory deficits. Also observed are muscular and retinal degeneration, potentially due to oxidative stress.	Kurihara et al., 2000; Wood et al., 2005; Sano et al., 2006
UCHL5/UCH37	Reversibly associates with the 19S component of the proteasome and deubiquitinates proteasome substrates. *Uchl5* knockout mice die during development due to brain malformations. In non-neural tissue, may regulate TGF-β signaling.	Wicks et al., 2005; Al-Shami et al., 2010; Clague et al., 2013
**USP**		
USP2	Involved in regulating the sensitivity to light of the circadian system in mice by deubiquitinating the transcription factor BMAL1. This DUB is upregulated in high-grade gliomas. Pan-neuronal knockdown in *Drosophila* leads to reduced locomotion and earlier adult lethality.	Scoma et al., 2011; Tsou et al., 2012; Tao et al., 2013
USP5	Co-purifies with synaptic 26S proteasome from rat cortex. Recycles mono-Ub by hydrolyzing unanchored ubiquitin chains. Pan-neuronal knockdown in *Drosophila* causes reduced motility and earlier adult death.	Reyes-Turcu et al., 2009; Tai et al., 2010; Tsou et al., 2012
USP7	Largely functions by preventing the proteasomal degradation of proteins, including p53 and REST. Brain-specific knockout causes brain malformation and neonatal lethality in mice, due at least in part to p53-dependent mechanisms.	Li et al., 2002; Huang et al., 2011; Kon et al., 2011
USP8	Pan-neuronal knockdown in *Drosophila* is developmentally lethal.	Tsou et al., 2012
USP9X	Involved in neuronal fate specification and NMJ function. Evidenced to regulate the degradation of the neurodegenerative disease protein α-synuclein by deubiquitinating it. May function outside of the UPP.	Huang et al., 1995; Fischer and Overstreet, 2002; Rott et al., 2011
USP13	Co-purifies with synaptic 26S proteasome from rat cortex.	Tai et al., 2010
USP14	Associates reversibly with the 19S proteasome and deubiquitinates proteasome substrates. Primary role in the nervous system appears to be maintenance of mono-Ub for reuse. May deubiquitinate specific substrates, including the neurodegenerative proteins tau and ataxin-3 to suppress their degradation, and the Wnt signaling regulator Dishevelled to regulate its interaction with partners. Mutations in mice cause ataxia and lead to abnormal NMJ structure and function, which is suppressed by reintroducing mono-Ub.	Wilson et al., 2002; Chen et al., 2009, 2011; Lee et al., 2010a; Jung et al., 2013
USP18	Knockouts in mice cause tremors, loss of balance, convulsions, and premature death. Anomalies appear to be related to the Ub-like protein ISG15, which USP18 can de-conjugate.	Ritchie et al., 2002; Knobeloch et al., 2005
USP22	Its *Drosophila* ortholog regulates axonal projection of photoreceptor cells.	Yi and Ehlers, 2007
USP24	May be involved in PD susceptibility, according to gene linkage analysis.	Li et al., 2006
USP25	Overexpressed in human Down syndrome brains. Evidenced to prevent the proteasomal turnover of the AD-related protein APP.	Valero et al., 1999; Blount et al., 2012
USP30	Opposes parkin-mediated mitophagy by deubiquitinating mitochondria in mammalian cells. Knockdown in *Drosophila* dopaminergic neurons suppresses PD-like degeneration.	Bingol et al., 2014
USP33	Regulates axonal pathfinding during development by regulating the stability or surface exposure of the axonal guidance receptor Roundabout.	Yuasa-Kawada et al., 2009
USP34	Pan-neuronal knockdown in *Drosophila* is developmentally lethal.	Tsou et al., 2012
USP36	Pan-neuronal knockdown in *Drosophila* leads to reduced locomotion and earlier adult death.	Tsou et al., 2012
USP39	Glial-restricted knockdown in *Drosophila* is developmentally lethal.	Tsou et al., 2012
USP40	May be involved in PD susceptibility, according to gene linkage analysis.	Li et al., 2006
USP46	Mutations in this DUB in mice cause anomalies in circadian rhythm and behavioral abnormalities during stress. In *C. elegans*, this DUB regulates lysosomal degradation of glutamate receptors.	Tomida et al., 2009; Kowalski et al., 2011; Kowalski and Juo, 2012
USP47	Pan-neuronal knockdown in *Drosophila* leads to reduced locomotion and early adult death.	Tsou et al., 2012
USP54	Pan-neuronal knockdown in *Drosophila* is developmentally lethal.	Tsou et al., 2012
**MJD**		
Ataxin-3	Mutations in it cause the neurodegenerative disease SCA3. Evidenced to cooperate with several E3 Ub ligases. May assist with the degradation of various UPP substrates. Suppresses polyglutamine-dependent degeneration in *Drosophila*.	Costa Mdo and Paulson, 2012; Tsou et al., 2013
**OTU**		
OTUB1	Found in Lewy bodies in post-mortem PD brains.	Xia et al., 2008
OTUD4	Mutations in this DUB and the E3 Ub ligase RNF216 have been linked to syndromic hypogonadotropic hypogonadism, ataxia, and dementia in humans. Knockdown in zebrafish causes anomalous development of the eye, optic tectum, and cerebellum.	Margolin et al., 2013
Otulin/Gumby	Mutations in the murine version of this DUB, which cleaves linear Ub chains, cause anomalies in angiogenesis, neuronal, and craniofacial development as a result of perturbed Wnt signaling.	Rivkin et al., 2013
**JAMM**		
PSMD14	Stoichiometric subunit of the 19S proteasome. Deubiquitinates substrates at the proteasome. Pan-neuronal knockdown in *Drosophila* leads to lethality during development.	Verma et al., 2002; Yao and Cohen, 2002; Clague et al., 2013
EIF3H	Pan-neuronal knockdown in *Drosophila* is developmentally lethal.	Tsou et al., 2012
AMSH	Knockout mice die young. Neuronal loss is observed in the cerebral cortex and the hippocampus.	Ishii et al., 2001; Suzuki et al., 2011

## UPP-related DUBs in the nervous system

### Proteasome-associated DUBs

#### PSMD14

This metallo-protease belongs to the JAMM sub-family of DUBs. It is also known as POH1. PSMD14 is a constitutive subunit of the proteasome, a member of the 19S regulatory complex, where it neighbors the ubiquitin receptor Adrm1 (Beck et al., 2012). Its reported role is to deubiquitinate proteins at the proteasome before they are degraded. By removing Ub chains *en bloc* from substrates, PSMD14 plays an important role in facilitating protein degradation, as well as in Ub recycling. Once poly-Ub is removed from substrates by PSMD14 it is disassembled by other DUBs, for example USP5 (Clague et al., 2013; Liu and Jacobson, 2013).

PSMD14 and its role at the proteasome were uncovered through studies in yeast that examined how deubiquitination and degradation of a substrate are coupled. In yeast, PSMD14 is known as Rpn11. Through the use of an unfolded, ubiquitinated model substrate, it was shown that deubiquitination occurs after a protein is bound by the proteasome. Mutating Ub so that it can no longer be cleaved by DUBs leads to a significant reduction of substrate degradation. Other studies showed that substrate deubiquitination at the proteasome is insensitive to molecules that block cysteine proteases, whereas a zinc chelator blocks deubiquitination, suggesting a metallo-enzyme. Mutations in Rpn11 stabilize UPP substrates and are lethal in yeast. These and other studies pointed to Rpn11 as the DUB functioning at the proteasome, bridging substrate deubiquitination and degradation (Verma et al., 2002; Yao and Cohen, 2002).

Work conducted in mammalian cell culture implicated PSMD14 in maintaining the post-mitotic status of neurons. RNAi-mediated knockdown of PSMD14 in mouse midbrain and cortical cultures leads to *de novo* synthesis of DNA and an increase in apoptosis (Staropoli and Abeliovich, 2005). The mechanism behind these observations is unclear, but these data suggest a critical role for UPP in preventing neurons from re-entering mitosis. Perhaps disturbance of the UPP (from the perspective of PSMD14) triggers a general cellular stress response that ultimately leads to neuronal death. Alternatively, the UPP could be involved in the continuous turnover of specific factors that induce mitotic re-entry. Highlighting the importance of PSMD14 to the nervous system, RNAi-mediated knockdown of its ortholog in neurons in *Drosophila* causes larval lethality (Tsou et al., 2012).

PSMD14 is one of very few DUBs with a specific function, in this case, the removal of poly-Ub chains in a single swoop from substrates at the proteasome. As it will become clear through the remainder of this review, it is difficult to categorize most DUBs as operating in only one step, or acting on only one substrate, pathway, tissue, or organ. Perhaps PSMD14 is an exception because of its continuous presence at the 19S subunit, where it is precluded from interacting with other potential partners. Most other DUBs do not appear to reside in a single constitutive molecular complex and can thus conduct different functions depending on their interactions.

#### UCHL5

This DUB is a member of the UCH sub-family and is also known as UCH37. Unlike PSMD14, UCHL5 is not a constitutive subunit of the proteasome but associates with it by binding to the Ub receptor Adrm1 on the 19S subunit (Lee et al., 2010a,b). The proposed role for this DUB at the proteasome is one of poly-Ub chain trimming by hydrolyzing Ub chains at their distal end and releasing mono-Ub for re-utilization (Yao et al., 2006). UCHL5 deubiquitinating activity has been shown to increase once it is bound to the proteasome (Qiu et al., 2006; Yao et al., 2006). Based on more recent work, UCHL5 stimulates the activity of the proteasome by regulating ATP hydrolysis and 20S gate opening (Peth et al., 2009, 2013a,b).

Studies in a *Uchl5*-null mouse line showed that this protease is important for brain development. *Uchl5* knockout embryos have malformations in various brain areas, including the telencephalon, mesencephalon, and metencephalon (Al-Shami et al., 2010). The reasons for these specific anomalies are not known. Other proteasome-associated DUBs, such as USP14, which is discussed next and which also processes chains to yield mono-Ub, appear unable to compensate for the loss of UCHL5, indicating a non-redundant role for this DUB in the brain.

#### USP14

USP14 belongs to the USP sub-family of DUBs. It reversibly associates with the 19S proteasome, where it is responsible for trimming Ub chains on substrates that are destined for degradation and thus recycling mono-Ub. The catalytic activity of USP14 is enhanced by its binding to the proteasome several hundred-fold (Lee et al., 2010a,b), suggesting that this DUB needs the proteasome in order to conduct functions that require its protease activity.

Depletion of USP14 alone enhances proteasome activity, whereas co-depletion with UCHL5 inhibits the proteasome (Koulich et al., 2008), indicating some cross-dependence for these DUBs during protein degradation. Critical information on the function of USP14 has come from yeast studies, where depletion of Ub leads to an upregulation of its ortholog, Ubp6, which restores Ub balance (Hanna et al., 2006). Not all functions of USP14 at the proteasome depend on its catalytic activity: binding of USP14 to poly-Ub chains and unfolded peptides results in opening of the gates of the 20S proteasome and stimulation of the ATPase activity of the proteasome (Peth et al., 2009, 2013a,b). Collectively, these findings implicated USP14 with keeping proteasomal activity under check.

A role for USP14 in the nervous system was first demonstrated by the identification of a recessive mutation in the *Usp14* gene, which causes ataxia in the *ax^J^* mouse line. Homozygous *ax^J^* mice suffer from progressive motor impairment including ataxia and tremor, reduced body and brain mass, paralysis and death by 2 months of age. The *ax^J^* mutation results from the insertion of an intracisternal-A particle into intron 5 of *Usp14*, leading to ~95% lower levels of USP14 protein in the brain of homozygous mice (Wilson et al., 2002; Anderson et al., 2005). In contrast to many mouse models of neurodegenerative diseases, *ax^J^* mice do not show neuronal loss or abnormal protein accumulation (Wilson et al., 2002). Instead, *ax^J^* mice have reduced mono-Ub levels, particularly in synaptosomal fractions (Anderson et al., 2005; Chen et al., 2009, 2011). The introduction of Ub in neurons in an *ax^J^* background largely suppresses the phenotype (Chen et al., 2011), indicating that a primary role of USP14 in the central nervous system is Ub homeostasis.

USP14 is critically important at the neuromuscular junction (NMJ). Recordings in *ax^J^* mice reveal defective release of the neurotransmitter acetylcholine (ACh) at the NMJ, and loss of USP14 leads to developmental anomalies at the pre- and post-synaptic terminals of the NMJ (Chen et al., 2009). The NMJs of *ax^J^* mice are swollen and poorly arborized, with aberrant nerve terminals and an immature morphology of ACh receptor clusters. These changes correlate with loss of mono-Ub, and are corrected by exogenous expression of Ub in neurons (Chen et al., 2009, 2011).

The ataxia phenotype in the *ax^J^* mice suggests that the cerebellum is negatively affected by nearly absent USP14. A potential role for this DUB in regulating the activity of cerebellar Purkinje cells has been reported. In *ax^J^* mice, Purkinje cells have increased cell surface expression of GABA_A_ Receptors (GABA_A_R) in extrasynaptic regions, with a concomitant increase in inhibitory GABAergic currents that effectively reduces cerebellar output (Lappe-Siefke et al., 2009). GABA_A_R is ubiquitinated in cells and USP14, which interacts directly with this receptor, may deubiquitinate it (Lappe-Siefke et al., 2009). Generally speaking, mono-ubiquitination controls recycling of various receptors to and from the cell membrane, including GABA_A_R (Saliba et al., 2007). Consequently, USP14 deficiency may cause ataxia in the *ax^J^* mice in part by perturbing the turnover and/or cell surface distribution of GABA_A_R. Still, since it is not clear that USP14 directly deubiquitinates GABA_A_R, it is also worth considering that this DUB may regulate the receptor indirectly. After all, USP14 is a sluggish protease outside of the proteasomal context, leading one to wonder whether it can deubiquitinate GABA_A_R in the absence of factors that enhance its catalytic activity.

Because of the early lethality phenotype in the *ax^J^* mice, it has been difficult to investigate whether USP14 is important in adults. Recently, another mutation in *Usp14* was identified (Marshall et al., 2013). This mutation, *nmf375*, leads to ~95% reduction in USP14 protein levels when homozygous, similar to the reduction observed in *ax^J^* mice. However, unlike *ax^J^* homozygous mice, ones carrying two copies of *nmf375* do not present with phenotypes early in life. Deterioration of motor performance is observed around 12 months of age (*ax^J^* mice die by 2 months) and is associated with mono-Ub depletion (Marshall et al., 2013). These data indicate that USP14 plays an important role not only for NMJ development, but also for its maintenance. They also highlight the importance of genetic modifiers: mutations that lead to similar reduction in USP14 protein levels in different genetic backgrounds have markedly different phenotypic onset and progression. Placing the *nmf375* into the same genetic background as the *ax^J^* leads to a dramatically earlier phenotype, even more severe than *ax^J^* (Marshall et al., 2013). Perhaps future genetic analyses will identify factors that modulate the phenotype caused by this UPP-related DUB. Could these modifiers be other DUBs related to the UPP, or are they E3 ligases that counteract USP14 function?

Studies from yeast, mammalian cell culture and mice together indicate that USP14 plays an important role in recycling mono-Ub by functioning at the proteasome. Other studies have presented the possibility that this DUB has specific substrates, potentially outside of the UPP. As described later, there is even evidence from cell culture that USP14 prevents the degradation of some ubiquitinated substrates at the proteasome, adding further complexity to the functions of this protease (see the Section on “The use of DUBs for therapeutic purposes”). Future work may find distinct binding partners of USP14 that depend on cell type, the state of neuronal activity, and on sub-cellular localization. In turn, these partners may dictate the precise function of USP14 as a DUB, or even as a scaffolding protein.

### DUBs that function in conjunction with the UPP

#### UCHL1

The first reported DUB with a neuronal function, UCHL1 is a member of the UCH sub-family of DUBs. The catalytic area of UCHL1 has a loop positioned over the active site, which limits the size of Ub adducts that can be processed by it to small peptides (Johnston et al., 1999; Das et al., 2006). Based on *in vitro* biochemical reactions, structural data and observations from animal studies, UCHL1 is proposed to function largely by maintaining a stable pool of mono-Ub for use in ubiquitination reactions (Clague et al., 2013). Newly translated Ub contains amino acids following the terminal glycine residue that is used for isopeptide bond formation. UCHL1 can cleave off these additional amino acids in order to expose the final glycine of Ub for conjugation. UCHL1 can also help maintain mono-Ub by reversing accidental modifications that can form during Ub activation (Larsen et al., 1998).

UCHL1 was first described in Aplysia, where its ortholog is known as Ap-UCH (Hegde et al., 1997). This protease was found to have a role in synaptic plasticity because it was one of the genes that was markedly upregulated in sensory neurons following LTF (long-term facilitation) and LTD (long-term depression). Inhibition of Ap-UCH induction or blockage of its function inhibits synaptic plasticity (Chain et al., 1995; Hegde et al., 1997; Fioravante et al., 2008).

UCHL1 is among the most abundant proteins in the brain, by some estimates reaching 1–2% (Jackson and Thompson, 1981; Doran et al., 1983; Wilkinson et al., 1992). Similar to Ap-UCH in Aplysia, mammalian UCHL1 is linked to synaptic function. Studies of mouse knockouts of the *Uchl1* gene indicate that this DUB is important for the structure and function of the NMJ. *Uchl1* knockout mice develop normally, but die prematurely after a period of spasticity and paralysis. At the level of the NMJ, *Uchl1* knockouts show a significant decrease in the release of ACh from the synaptic terminal (Chen et al., 2010), which could be due to perturbed Ub-dependent pathways as a result of decreased Ub recycling. This reduction in content release is accompanied by hindered synaptic plasticity, nerve terminal retraction and axonal degeneration (Chen et al., 2010). Supportive evidence for a role for UCHL1 at the synapse also comes from the *gad* (gracile axonal dystrophy) mouse line, which has an intragenic *Uchl1* deletion (Saigoh et al., 1999). Similar to the *Uchl1* knockout mice, ones homozygous for *gad* present with a dying-back type of axonal degeneration. Lastly, studies in rat hippocampal slices also collected evidence that UCHL1 is important for synaptic plasticity by maintaining mono-Ub. Increased UCHL1 activity leads to higher levels of mono-Ub, whereas pharmacological inhibition of this DUB has the opposite effect and is associated with anomalous synaptic spine structure (Cartier et al., 2009). Importantly, anomalies at the synaptic level during UCHL1 inhibition are rescued by the introduction of mono-Ub (Cartier et al., 2009). Together, these data indicate that a primary role for UCHL1 in the nervous system is to maintain mono-Ub available for utilization during inter-cellular communication. This pool of mono-Ub could be utilized by the UPP as well as other types of cellular pathways and processes, such as gene transcription, receptor internalization, autophagy, etc.

UCHL1 is important to the ageing nervous system, as highlighted by the connection of this DUB to age-related diseases, including Alzheimer's (AD) and Parkinson's (PD). Based on proteomic studies, UCHL1 is a major target of oxidative damage in AD and PD post-mortem human brains (Choi et al., 2004). UCHL1 protein levels are reduced in the hippocampus of a transgenic mouse model of AD that has learning deficits and impaired LTF (Gong et al., 2006). Similarly, soluble UCHL1 levels are decreased in post-mortem AD brains, potentially due to its sequestration in neurofibrillary tangles (Setsuie and Wada, 2007). Introduction of UCHL1 in transgenic AD mice and in cultured cells alleviates cognitive defects and restores synaptic plasticity in a manner dependent on its catalytic activity (Gong et al., 2006). These and other findings support previously mentioned data that UCHL1 is important at synapses, and suggest that increased UCHL1 activity could counteract certain symptoms in AD.

Other work proposes that UCHL1 has specific substrates and may increase UPP-dependent degradation of the β-site amyloid precursor protein (APP) cleaving enzyme 1 (BACE1). BACE1 sequentially cleaves APP into amyloid ß (Aß) protein, which is a major component of neuritic plaques, a hallmark of AD. Pharmacological inhibition of UCHL1 in cultured cells leads to an increase in BACE1 protein levels, whereas an increase in UCHL1 levels has the opposite effect and is associated with reduced levels of Aß protein. Studies in cultured cells are supported by investigations in *gad* mice. Measuring hippocampal levels of BACE1 by western blotting shows an increase in its protein levels in *gad* mice compared to wild type counterparts (Zhang et al., 2012). Whether UCHL1, with its physically constrained catalytic site, can deubiquitinate BACE1 and how this would accelerate its degradation is presently unclear. Another possibility is that reduced mono-Ub levels as a result of UCHL1 perturbation inhibits the UPP more generally, which in turn impacts BACE1 stability.

In relation to PD, a missense mutation in UCHL1 (I93M) was described in 1998 as the cause of dominantly-inherited PD in a family (Leroy et al., 1998). In *in vitro* reconstituted assays, UCHL1^I93M^ was found to have reduced DUB activity (Nishikawa et al., 2003), thus it was initially hypothesized that PD could be due to partial loss of UCHL1 activity. However, mice lacking UCHL1 do not develop neurodegenerative hallmarks of PD, such as loss of dopaminergic neurons, indicating that PD in the I93M family might not be due to reduced UCHL1 activity, but could result from a gain-of-function. To examine this latter possibility, transgenic mice were generated expressing *UCHL1^I93M^*. These mice show loss of nigral dopaminergic neurons, as is characteristically seen in PD, and develop Ub- and UCHL1-positive inclusions, although not Lewy Bodies, which are the histopathological hallmark of PD (Setsuie and Wada, 2007; Setsuie et al., 2007; Tan and Skipper, 2007). According to these results, UCHL1^I93M^ could cause neurodegeneration through a gain-of-function mechanism. The molecular process through which this mutation would impact UCHL1 cellular function, localization or other properties is unclear.

Another report that linked UCHL1 to PD presented evidence that the DUB activity of a farnesylated, membrane-bound form of this protease rescues the PD protein, α-synuclein, from lysosomal degradation (Liu et al., 2009). α-synuclein is implicated in the buildup of aggregated structures in patient brains, and this buildup is believed to be one cause of death of dopaminergic neurons in PD (Kruger et al., 1998; Athanassiadou et al., 1999; Tan and Skipper, 2007). If it is confirmed *in vivo* that farnesylated UCHL1 has a significant role in the turnover of α-synuclein, it would suggest this DUB as a potential therapeutic target for PD. Severing UCHL1 from the membrane might increase the degradation of α-synuclein, effectively reducing levels of this aggregation-prone protein and alleviating neuronal stress. As it will be discussed later, α-synuclein can be degraded by the proteasome as well as through autophagy. Each type of degradation rests on Ub-dependent processes that are controlled by DUBs such as UCHL1 and USP9X (see below). Consequently, targeting the stability of this disease-linked protein for therapy will require the consideration of various regulatory processes.

Lastly, in 2013 three siblings from a Turkish family were discovered with a recessive, missense mutation in the Ub-binding domain of UCHL1 (mutation E7A), which leads to markedly reduced catalytic activity of this DUB *in vitro*. Patients from this family show an early-onset progressive neurodegenerative syndrome that includes cerebellar ataxia, spasticity, blindness, and nystagmus (Bilguvar et al., 2013). These symptoms are different from those of PD patients, including the ones who carry the I93M mutation. Such phenotypic variability could be due to different effects of the E7A and I93M mutations on the activity and interactions of UCHL1, and might be compounded by other genetic differences between the families.

As we near the end of this section, it is important to note that mutations in two different DUBs, USP14 and UCHL1, which function at least by maintaining mono-Ub, cause problems at the NMJ and neurological defects in mice. Does this mean that they share duties at the molecular, cellular, and tissue level? These DUBs are linked through mono-Ub: *Uchl1* transcription is upregulated in *ax^J^* mice and, vice versa, *Usp14* transcription is increased in *Uchl1*-deficient mice (Walters et al., 2008), suggesting a molecular circuitry that controls the expression of these genes in response to depleted mono-Ub. However, this upregulation at the transcription level does not lead to suppression of the phenotype, or restitution of mono-Ub levels. Neurological symptoms differ among *Uchl1*- and *Usp14*-deficient mice, which could be due to differences in genetic background. As discussed above, genetic background can have a profound effect on symptoms and progression of ataxia in *Usp14*-mutant mice (Marshall et al., 2013). Another reason why mutations in USP14 and UCHL1 lead to different phenotypes could stem from partially non-redundant functions. Perhaps there are specific cellular processes (or substrates), populations of neurons (or glia), or stages of development for which one DUB is more critical than the other. While USP14 and UCHL1 have similar effects on the levels of mono-Ub available in neurons, they likely also play divergent roles in other aspects of neuronal homeostasis that remain to be elucidated.

#### USP7

A member of the USP sub-family of DUBs, USP7 is also known as HAUSP. It has gained attention because it is viewed as a potential therapeutic target for malignancies. Based on numerous studies, USP7 functions closely with the UPP, where it opposes the proteasomal degradation of various substrates, including the E3 Ub ligase murine double mutant 2 (mdm2) and its substrate, p53, a tumor suppressor that causes cell cycle arrest and apoptosis (Clague et al., 2013). USP7 deubiquitinates both mdm2 (which enhances p53 degradation) and p53 (which inhibits p53 degradation) (Li et al., 2002, 2004). It is the interplay of p53 and USP7 that may be critical for the nervous system. Through a brain-restricted knockout strategy for *Usp7* in mice, it was shown that this DUB is essential: mice lacking USP7 in the brain die soon after birth and have anomalous brain development, attributed in part to p53 protein stabilization as a result of increased mdm2 turnover in the absence of USP7 (Kon et al., 2011). p53-independent mechanisms may also be involved in neonatal lethality, because inactivation of p53 through a knockout strategy fails to fully rescue lethality in the absence of USP7 (Kon et al., 2011).

Another potential role for USP7 in the nervous system is the regulation of the repressor element 1-silencing transcription factor (REST), which inhibits neural cell differentiation. Knockdown of USP7 in neuronal progenitor cells leads to a decrease in the levels of REST protein. USP7 and REST co-immunoprecipitate, and USP7 knockdown results in higher levels of poly-Ub REST, suggesting that USP7 controls REST by deubiquitinating it (Huang et al., 2011). During differentiation, REST is targeted for proteasomal degradation by multiple E3 ligases. The levels of one such ligase, ß-TrCP, increase during neuronal differentiation, when levels of USP7 and REST are lower. When both USP7 and ß-TrCP are knocked down, an intermediate level of ubiquitinated REST is observed compared to its levels in cells where only one protein is targeted (Huang et al., 2011). According to these results, ß-TrCP and USP7 counterbalance each other in regulating the stability of REST. This interaction between USP7 and ß-TrCP is reminiscent of the USP7/mdm2 exchange with respect to p53. It is presently unclear whether p53-independent anomalies in brain development as a result of *Usp7* knockout are due to perturbation in REST signaling. Collectively, these studies suggest at least two major pathways that can be regulated by USP7 in the nervous system: neuronal differentiation and cell viability.

USP7 may also regulate other processes in the brain, including in some neurodegenerative diseases. USP7 interacts with the gene transcription protein ataxin-1 (Hong et al., 2002), mutations in which cause the age-related neurodegenerative disease Spinocerebellar Ataxia Type 1 (Williams and Paulson, 2008). The physiological implications of this interaction are uncertain, but suggest the possibility of USP7 roles in stages following nervous system development. Lastly, recent studies indicated that the above-mentioned transcription factor, REST, is positively involved in normal ageing, and its loss may be implicated in AD and mild cognitive impairment (Lu et al., 2014). Considering the role of USP7 in REST turnover during development, one can extrapolate that this DUB may be neuroprotective in the ageing brain by suppressing REST degradation.

#### USP9X

Another member of the USP sub-family of DUBs implicated in the nervous system is USP9X. The *Drosophila* ortholog of USP9X, Faf, has been linked to the development of fly eyes and the NMJ (Fischer-Vize et al., 1992; Diantonio et al., 2001). Faf overexpression in *Drosophila* results in NMJ overgrowth, including an increase in synaptic span and number of synaptic boutons. Faf genetically interacts with the E3 ligase Highwire (Hiw), because loss of function in Hiw results in a phenotype similar to Faf overexpression (Diantonio et al., 2001). These findings implicate yet another E3 ligase/DUB pair balancing each other's functions, although the molecular events downstream of Faf and Hiw remain to be uncovered.

In fly eyes, Faf prevents over-neuralization during development. Fruit flies lacking this DUB or expressing a version that is catalytically inactive have supernumerary photoreceptors due to aberrant differentiation of cells that normally acquire non-neural fates (Fischer-Vize et al., 1992; Huang et al., 1995). The fate of these cells is specified through Notch-Delta signaling. Faf genetically interacts with Liquid facets (Lqf), an ortholog of mammalian epsins involved in endocytosis (Cadavid et al., 2000; Chen et al., 2002). Lqf is important for Delta internalization during Notch signaling and thus is a critical component of cell fate specification. In *Drosophila*, decreasing Lqf levels enhances the supernumerary photoreceptor phenotype of Faf mutants, while increasing Lqf levels renders Faf unnecessary (Cadavid et al., 2000; Chen et al., 2002). Ubiquitination of Lqf is stabilized in Faf-less eyes, Faf and Lqf co-immunoprecipitate, and Lqf protein levels are lower in Faf-null mutants (Chen et al., 2002). These findings lead to the conclusion that Faf regulates the ubiquitination status and stability of Lqf: in the absence of Faf, ubiquitinated Lqf could be targeted for proteasomal degradation. It is possible that Lqf ubiquitination also modulates its activity. The ability of human epsins to interact with partners can be regulated by mono-ubiquitination (Nijman et al., 2005; Yi and Ehlers, 2007). Consequently, Faf-dependent deubiquitination of Lqf may control both its function and stability. The human version of Faf, USP9X, also interacts with the Lqf ortholog, epsin-1, and co-localizes with it at the synapse (Chen et al., 2003). Since RNAi against USP9X stabilizes ubiquitinated epsin-1 in cultured cells (Chen et al., 2003), we can infer an evolutionarily conserved role for USP9X in regulating epsin-1 through deubiquitination.

USP9X has been implicated in two neurodegenerative disorders: PD and Diffuse Lewy Body Disease (DLBD). USP9X expression is altered in a mouse model of PD (Zhang et al., 2010), and a portion of USP9X localizes to Lewy Bodies in PD and DLBD (Rott et al., 2011). Supporting the possibility of a role for USP9X in disease, studies have linked this DUB to the stability of α-synuclein, which has been implicated in the etiology of both PD and DLBD. USP9X is reported to regulate the cellular turnover of α-synuclein by deubiquitinating it. RNAi targeting USP9X leads to higher levels of mono-ubiquitinated α-synuclein in cultured cells and these two proteins co-immunoprecipitate from cultured cells and rat brain lysates. Higher levels of mono-ubiquitinated α-synuclein are not well tolerated by cells (Rott et al., 2011), indicating that USP9X activity can protect against toxicity from this protein. By deubiquitinating α-synuclein, USP9X seems to specify the degradative pathway through which this disease protein is disposed: UPP or autophagy. Unlike other proteins, α-synuclein reportedly only requires mono-ubiquitination to be degraded by the proteasome. However, upon deubiquitination by USP9X, α-synuclein is removed through autophagy (Rott et al., 2011). Perhaps this information on USP9X and α-synuclein can be used for therapeutics for PD and DLBD. Activators of USP9X are expected to reduce levels of mono-ubiquitinated α-synuclein—which is toxic to cells—and to increase its degradation through autophagy, resulting in a neuroprotective effect.

As mentioned above, membrane-bound, farnesylated UCHL1 can also regulate α-synuclein in cell culture by protecting it from lysosomal degradation through a mechanism that remains to be elucidated (Liu et al., 2009). It is tempting to speculate that UCHL1 prevents the lysosomal degradation of α-synuclein by directly deubiquitinating it, but this model would not necessarily fit with the data summarized above on USP9X and the degradation of this disease protein. Potentially, UCHL1 regulates α-synuclein indirectly by controlling other proteins that dictate its turnover, including perhaps USP9X. Ultimately, therapeutic approaches based on the role of USP9X or UCHL1 on α-synuclein need to consider how these DUBs might affect each other's regulatory effect on this aggregation-prone protein.

#### Ataxin-3

Ataxin-3 is a member of the MJD sub-family of DUBs. It first received attention because it is the disease protein in the neurodegenerative disorder Spinocerebellar Ataxia Type 3 (SCA3), also known as Machado-Joseph Disease. SCA3 is an age-related disease that belongs to the family of triplet repeat disorders, more specifically the polyglutamine repeat-related diseases that include Huntington's, Spinobulbar Muscular Atrophy, Dentatorubral-Pallidoluysian Atrophy and five more SCAs (SCA1, 2, 6, 7, and 17) (Todi et al., 2007). SCA3, which is believed to be the most common dominantly inherited ataxia in the world, is a progressive ataxia accompanied by dystonia, dysarthria, spasticity, rigidity, ophthalmoparesis, dysphagia, and neuropathy. Pathology includes degeneration of cerebellar pathways and nuclei, pontine and dentate nuclei, substantia nigra, globus pallidus, cranial motor nerve nuclei, and anterior horn cells. SCA3 is caused by a CAG repeat expansion in the gene *ATXN3*, which encodes the DUB ataxin-3 (Costa Mdo and Paulson, 2012).

Based on *in vitro* biochemistry, cell-based studies and *in vivo* work in mice, *C. elegans* and *Drosophila*, ataxin-3 seems to function in the UPP (Matos et al., 2011; Costa Mdo and Paulson, 2012). Under some circumstances, ataxin-3 may enhance the degradation of some proteasome substrates (e.g., in Endoplasmic Reticulum (ER)-Associated Degradation, Wang et al., 2006, and in relation with the Ub ligase CHIP, Scaglione et al., 2011), while under other conditions it may decelerate proteasomal degradation of other proteins (Zhong and Pittman, 2006). Since ataxin-3 interacts with several E3 Ub ligases (CHIP, E4B, Hrd1, Parkin) and with the proteasome-associated proteins hHR23A, hHR23B, and VCP/p97 (Costa Mdo and Paulson, 2012), this DUB may regulate the fate of numerous UPP substrates. The precise outcome of ataxin-3 function—increased or decreased stability of proteins—most likely depends on the protein partners with which it interacts.

Through a series of biochemical assays, it was shown that the E3 Ub ligase CHIP and its E2 partner Ubch5C attach long poly-Ub chains onto model substrates. Ataxin-3 cooperates with CHIP/Ubch5C to restrict or edit the length of these poly-Ub species. This collaboration serves to enhance, rather than prevent, proteasomal degradation of CHIP substrates such as iNOS (Scaglione et al., 2011), probably because very long poly-Ub can hinder proteasomal activity (Kim et al., 2007, 2009). Thus, in contrast to what was described above for USP7 and mdm2, which can oppose each other's activities, ataxin-3 and CHIP appear to work together to enhance the turnover of at least some proteins. Such collaborative interactions between ligases and DUBs could be common, because a proteomic study of DUBs identified numerous E3 Ub ligases co-precipitating with these proteases (Sowa et al., 2009).

There is also evidence that ataxin-3 can suppress the degradation of some UPP substrates in cells. Work conducted on ER-Associated Degradation indicates that this DUB deubiquitinates some misfolded proteins synthesized in the ER, thus preventing their proteasomal degradation. Ataxin-3 appears to perform this function in relation with the proteasome-associated protein VCP/p97 (Zhong and Pittman, 2006), although it has not been ruled out that ataxin-3 may also directly oppose the function of ER ligases such as Hrd1 or AMFR.

Three different knockout mouse lines for *atxn3* are viable and appear to live normal lives, indicating that *atxn3* is a non-essential gene (Schmitt et al., 2007; Reina et al., 2010; Switonski et al., 2011). However, this DUB might be required under certain physiological conditions. For example, mouse embryonic fibroblasts that lack ataxin-3 fair poorly during heat shock (Reina et al., 2010, 2012). Also, exogenous ataxin-3 suppresses toxicity from polyglutamine proteins in *Drosophila* (Warrick et al., 2005; Tsou et al., 2013). Whether ataxin-3 has a protective role *in vivo* in mice is less clear. In one study, evidence was presented that wild type ataxin-3 suppresses pathology from its disease-causing version in mouse models of SCA3 (Cemal et al., 2002). But, in a recent publication lack of ataxin-3 did not seem to enhance pathology in a Huntington's Disease mouse model (Zeng et al., 2013). As this latter study did not test whether higher levels of ataxin-3 had a protective effect, or whether catalytically inactive ataxin-3 worsened the HD phenotype, it remains to be clarified whether this DUB has a neuroprotective effect in mammals.

The molecular mechanisms that lead to polyglutamine-dependent neurodegeneration in SCA3 are uncertain. Based on *in vitro* studies, K48- and K63-linked poly-Ub binding and cleaving capabilities of ataxin-3 are not affected by expansions in the polyglutamine repeat (Burnett et al., 2003; Winborn et al., 2008; Todi et al., 2009), although its ability to cleave K27- and K29-linked poly-Ub is enhanced in the disease-causing version (Durcan et al., 2011). In cells, polyglutamine-expanded ataxin-3 is less efficient at reducing levels of ubiquitinated species, suggesting that some of its cellular roles are affected by expansion (Winborn et al., 2008). Supporting the notion that polyglutamine expansion alters ataxin-3 function, a disease-causing version of this protease targets the E3 Ub ligase parkin for degradation through autophagy, unlike wild type ataxin-3 (Durcan et al., 2011). Based on studies from other polyglutamine proteins such as ataxin-1 (Lam et al., 2006), polyglutamine repeat expansion may lead to both a partial loss-of- and a toxic gain-of-function for ataxin-3, although this remains to be elucidated. Since ataxin-3 appears to be non-essential in mice, perhaps one effective therapeutic route for SCA3 is to get rid of the protein, potentially without the need to discriminate between normal and pathogenic forms.

### The use of DUBs for therapeutic purposes

The UPP has attracted particular attention from a therapeutic point of view because of its critical role in protein quality control and, consequently, in regulating numerous cellular pathways. Different components of the UPP have been targeted for therapy, most commonly the proteasome itself, although not directly for neurological diseases. Therapies focusing on the proteasome have been developed and have shown promise in the clinic. Bortezomib (PS-341/Velcade®), which inhibits the activity of the 20S component, is a treatment option for multiple myeloma and mantle cell lymphoma (Clague et al., 2013). Another inhibitor, carfilzomib (PR-171/Kyprolis®), was generated to treat patients desensitized to bortezomib. Although both of these inhibitors show preference for killing cancer cells, inhibiting the proteasome itself is largely nonspecific and leads to various undesirable side effects (Colland, 2010; Mujtaba and Dou, 2011). Focusing on more specific steps and components upstream of the proteasome could provide desired therapeutic effects without perturbing general proteostasis. This is where DUBs might be particularly useful. Structural work has revealed significant differences in the catalytic areas of DUBs (Clague et al., 2013) that could be utilized to design DUB-specific inhibitors. High-throughput screens have identified small molecule inhibitors for USP7 and UCHL1 (Colland, 2010; Todi and Das, 2012), although the efficacy and overall effect of these inhibitors in intact animals, particularly in the nervous system, is not clear.

One DUB that has been targeted therapeutically for the nervous system is USP14. As discussed earlier, this DUB associates reversibly with the proteasome and is proposed to be important in maintaining a pool of mono-Ub for utilization. USP14 may also function by rescuing certain proteins destined for degradation by deubiquitinating them at the proteasome. Cells that do not express USP14 have enhanced clearance of several disease-related proteins, including tau (related to AD), TDP-43 (AD and Amyotrophic Lateral Sclerosis), and ataxin-3 (SCA3, discussed above) (Lee et al., 2010a). This finding suggests that inhibiting the catalytic activity of USP14 may have therapeutic benefits. It is not clear what cellular conditions or substrate particularities dictate the ability of USP14 to rescue some substrates from degradation.

A screen was conducted to identify inhibitors of USP14, and one particularly promising compound was isolated, 1-[1-(4-fluorophenyl)-2,5-dimethylpropyl-3-yl]-2-pyrrolidin-1-ylethanone (abbreviated as IU1), with remarkable specificity against the catalytic activity of USP14 (Lee et al., 2010a). Treatment with IU1 enhances the clearance of disease-linked proteins in cultured cells in a manner that is proteasome dependent (Lee et al., 2010a). A series of *in vitro* and cell-based experiments indicated that trimming of poly-Ub chains by USP14 has an antagonistic effect on protein degradation by the proteasome: proteasomes incubated with USP14 show decreased degradation of a model ubiquitinated substrate compared to inactive USP14, which does not inhibit proteasomal degradation (Lee et al., 2010a). By inhibiting the deubiquitinating activity of USP14, IU1 would presumably prevent the rescue of some ubiquitinated neurotoxic proteins at the proteasome (such as tau and ataxin-3), leading to their degradation. It remains to be determined if IU1 can prevent neurodegeneration *in vivo*, particularly in light of more recent work, which found that overall levels of ataxin-3 and tau were not different in USP14-deficient mice (Jin et al., 2012). These potential discrepancies in findings from cultured cells and intact animals could result from different handling of ataxin-3 and tau *in vitro* vs. *in vivo*. Acute inactivation of USP14 could increase the degradation of some disease proteins by the proteasome without perturbing Ub homeostasis, whereas chronic inactivation could cause an overall disturbance in mono-Ub availability that leads to compensatory mechanisms of protein turnover (such as lysosomal-dependent degradation). Additonally, inactivation or depletion of USP14 may not have beneficial effects in all neurodegenerative diseases. USP14 can reduce aggregates formed by mutant huntingtin (which causes Huntington's Disease) and can suppress cellular degeneration caused by this protein (Hyrskyluoto et al., 2014). The molecular mechanism through which USP14 has this neuroprotective effect in cells is not known, but it might involve: (1) mono-Ub availability for proper UPP function, (2) the deubiquitination of huntingtin aggregates, which are heavily ubiquitinated, for better recognition, access and degradation by the proteasome, as well as (3) changes in ER-Associated Degradation (Hyrskyluoto et al., 2014). Altogether, these findings stress the importance of a deep understanding of the UPP before targeting specific components for blanket therapeutics in neurodegenerative diseases.

Other studies have hinted at potential molecules that could be used to regulate DUB activities in the nervous system. A recent report that investigated the mechanism through which metal complexes inhibit proteasome function for cancer therapy found that copper pyrithione (CuPT) acts at two distinct steps: (1) by inhibiting the activity of the 20S component, and (2) by inhibiting USP14 and UCHL5 (Liu et al., 2014). CuPT might be a promising cancer therapeutic by inhibiting UPP and causing cellular death in malignant cells, although its utility in neurodegenerative diseases is doubtful. Inhibiting neuronal UPP would be detrimental to the nervous system and would exacerbate pathology. Still, CuPT might be an important experimental reagent to understand the function of USP14 and UCHL5 in the nervous system, especially if this complex can be modified so that its inhibitory roles on the 20S and USP14 and UCHL5 are dissociated. A molecule with such properties in fact exists: b-AP15, which inhibits the DUB activity of USP14 and UCHL5 (D'Arcy et al., 2011). To the best of our knowledge, neither compound has reportedly been utilized to investigate the nervous system.

## Conclusions

Understanding the functions of enzymes important for the removal of Ub from substrates targeted for UPP-dependent degradation provides a deeper appreciation of a basic cellular process essential to all eukaryotic cells. We hope to have made a convincing case that DUBs that function in conjunction with the UPP play critical roles in nervous system development, function, and disease.

The job of the UPP is to identify proteins that need to be degraded and selectively send them to be destroyed by the proteasome through the utilization of an identifier, in this case, ubiquitination. DUBs are among the various checkpoints that ensure the ubiquitination of correct substrates. Some such proteases, like ataxin-3, can function by editing the type of chain attached to a substrate in order to enhance the proteasomal degradation of a specific protein. Others, exemplified by USP7 and USP9X, can oppose the function of specific E3 Ub ligases, leading to the stabilization of some proteasomal substrates. DUBs such as UCHL1, UCHL5, and PSMD14 seem critical for mono-Ub homeostasis; others, like USP14, control the UPP through multiple, seemingly contradictory, activities: by recycling Ub for reuse, by increasing proteasome function, and sometimes by rescuing specific substrates from degradation (Figure 4).

As there are nearly 95 genes encoding DUBs in humans, one would think that there would be redundancy built into the functions of this family of proteases. However, studies in various animal models indicate that redundancy is not common among DUBs (Clague et al., 2013). Mutations or knockouts of different DUBs lead to distinct developmental anomalies or neurological disorders, supporting the idea that while some DUBs share duties, they also have roles in pathways, cells, tissues, organs, and stages of development that cannot be fully compensated by other members of their family.

While we have learned a tremendous amount about the UPP and the role of DUBs in it, much remains to be uncovered, particularly in relation to the nervous system. Each time a door is opened by new research, a spiraling corridor is revealed with more possibilities to consider and opportunities to explore. Details on UPP-related DUBs in specific neuronal circuits are limited. We lack an expression and localization atlas of DUBs during development and in adults, during increased periods of activity and in resting times, in different areas of the brain and spinal cord, in different types of neuronal and glial cells and in distinct sub-cellular compartments. Such information is critical if we are to understand why the perturbation of DUBs that are supposed to have somewhat related functions has different outcomes in intact organisms. Rewards from the continued study of DUBs and UPP in the nervous system are expected to be great, both in terms of comprehending basic mechanisms of neuronal physiology, and in understanding and treating diseases of the nervous system.

## Author contributions

Wrote Manuscript: Gorica Ristic, Wei-Ling Tsou, Sokol V. Todi. Prepared Figures: Gorica Ristic, Wei-Ling Tsou. Prepared Table: Gorica Ristic, Sokol V. Todi.

## Note

While this manuscript was under review and in production, further work was published on DUBs important to the nervous system, including reports on UCHL1 and its importance in the enteric nervous system (Coulombe et al., 2014) and in tau phosphorylation and AD pathogenesis (Zhao et al., 2014). We regret the inability to discuss this work as well as other reports in this review.

## Funding

This work was supported by R01 NS086778 from NINDS to Sokol V. Todi.

### Conflict of interest statement

The authors declare that the research was conducted in the absence of any commercial or financial relationships that could be construed as a potential conflict of interest.
